# Ochrobactrum Peritonitis: Case Report and Literature Review

**DOI:** 10.7759/cureus.10564

**Published:** 2020-09-21

**Authors:** Edward Medeiros, Kevin Tang, Susie Hu, Ankur Shah

**Affiliations:** 1 Department of Internal Medicine, Warren Alpert Medical School, Brown University, Providence, USA; 2 Division of Kidney Diseases and Hypertension, Rhode Island Hospital, Providence, USA; 3 Department of Internal Medicine, Rhode Island Hospital, Providence, USA

**Keywords:** peritoneal dialysis, peritonitis, end-stage kidney disease

## Abstract

End-stage kidney disease (ESKD) patients, including those on peritoneal dialysis (PD), are considered immunocompromised and at risk for opportunistic pathogens. Peritonitis is a major infectious PD complication with common causative pathogens, including gram-positive organisms such as coagulase-negative Staphylococcus species, Staphylococcus aureus more often than gram negative organisms. PD peritonitis is often secondary to suboptimal technique leading to contamination of the catheter site but can also be due to bacterial translocation from the bowel lumen or transient bacteremia after procedures; this makes identification of the causative organism crucial to optimal management of PD peritonitis. Ochrobactrum are glucose-non-fermentative, non-fastidious, motile gram-negative bacilli typically isolated in aqueous environments. Reported infections primarily occur in immunocompromised hosts with environmental exposure, including nosocomial contamination of fluids or indwelling catheters. We present only the seventh reported case of Ochrobactrum peritonitis in a 67-year-old PD patient secondary to poor technique, and review the literature for all prior cases. Although there have been no previous cases leading to bacteremia, three of the seven cases required removal of PD catheter.

## Introduction

Patients with end-stage kidney disease (ESKD), particularly those on peritoneal dialysis (PD), are considered immunocompromised and at risk for opportunistic pathogens. Peritonitis is a major infectious PD complication; common causative pathogens include gram-positive organisms such as coagulase-negative Staphylococcus species, Staphylococcus aureus*, *and Enterococcus. Gram-negative organisms are responsible for 15%-35% of cases, and failure to identify an organism is frequent [1-3]. PD peritonitis is often secondary to suboptimal technique leading to contamination of the catheter site but can also be due to bacterial translocation from the bowel lumen or transient bacteremia after procedures; proper culture technique is fundamental to identifying the causative organism [4]. Ochrobactrum anthropiis a gram-negative bacillus isolated from hospital and environmental water sources that can be pathogenic in humans [5]. We discuss a case of peritonitis secondary to Ochrobactrum anthropi, a rare bacterium, in a 67-year-old PD patient.

## Case presentation

A 67-year-old male with a history of ESKD on PD secondary to IgA nephropathy on continuous cycling peritoneal dialysis (CCPD) for four years presented to the hospital with fevers, nausea, abdominal pain, and generalized weakness for two days. History was also significant for hypertension (HTN) and deceased-donor kidney transplant (DDKT) with graft failure no longer on immunosuppression. He experienced an episode of PD-associated bacterial peritonitis one month prior to presentation with cultures speciating Pseudomonas aeruginosa. He completed a three-week course of oral ciprofloxacin. He had no other history of dialysis complications. His peritoneal effluent initially cleared with treatment and again became cloudy after one month.

Initial vital signs included temperature 36.7°C, pulse 106 beats per minute, blood pressure 176/104 mmHg, and oxygen saturation 98% on room air. Exam was significant for clear lung fields, no lower extremity edema, and diffuse abdominal tenderness to palpation. The peritoneal catheter exit site did not show any drainage or erythema.

A peritoneal effluent sample was found to have 347 nucleated white blood cells, 56% of which were neutrophils, and one red blood cell per milliliter. Peritonitis was diagnosed and treatment commenced with intraperitoneal (IP) vancomycin and cefepime, as well as oral fluconazole for fungal prophylaxis. Peritoneal effluent culture grew Ochrobactrum anthropi, sensitive to fluoroquinolones and carbapenems but resistant to cefepime. Antimicrobials were subsequently transitioned to oral ciprofloxacin and oral fluconazole for fungal prophylaxis, and he completed a three-week course with resolution of symptoms and peritoneal leukocytosis. The patient experienced a repeat episode of peritonitis six weeks after this with cultures again speciating Ochrobactrum and was transitioned to hemodialysis with removal of the PD catheter. He did not complete retraining in the interim period.

## Discussion

Ochrobactrum species* *are glucose-non-fermentative, non-fastidious, motile gram-negative bacilli typically isolated in aqueous environments. Reported infections primarily occur in immunocompromised hosts with environmental exposure, nosocomial contamination of sterile fluids, and/or indwelling catheter use [1]. In addition to Ochrobactrum bacteremia, cases of pneumonia, peritonitis, meningitis, and cystitis have also been described.

ESKD comprises both innate and adaptive immunity and confers increased risk for infection by opportunistic pathogens [6,7]. The mechanism of immunologic dysfunction is debated but may include impairment of Toll-like receptors, cytokine production, and malfunction of antigen presenting cells and T-lymphocytes [8].

Ochrobactrum infections have typically been seen in immunocompromised hosts, and we present the seventh case of Ochrobactrum peritonitis in a PD patient.

In any case of peritonitis, it is important to evaluate for improper technique and provide retraining to the patient. Technique lapses are known to increase the risk of peritonitis [9]. Studies evaluating patient technique find rates of technique lapse to be present in nearly 50% of patients [9,10]. This is particularly pertinent in those with Ochrobactrum peritonitis, as this pathogen is more likely more likely to occur from lapse in technique rather than from gastrointestinal translocation. Evidence on the efficacy of retraining is lacking as well as standardization of content and timing, with one randomized controlled trial limited by discontinuation in 77%-80% of subjects; however, this remains a best practice [11].

Cases are published in both continuous ambulatory PD and CCPD, with none of the published cases associated with bacteremia. Attempted treatments have included carbapenems, aminoglycosides, and fluoroquinolones. Three of seven cases required removal of the PD catheter and rate of peritonitis-related mortality was zero percent, suggesting the virulence of Ochrobactrum species is variable. In addition to the three patients requiring catheter removal, one patient presenting with fevers and hypotension had Ochrobactrum isolated from her peritoneal effluent without peritoneal leukocytes. Her symptoms resolved without antibiotics or further intervention, and she cleared the organism without therapy (Table 1). 

**Table 1:** Summary of reported PD peritonitis secondary to Orchrobactrum. CAPD, continuous ambulatory peritoneal dialysis; IP, intraperitone; TMP-SMX, trimethoprim-sulfamethoxazole; HD, hemodialysis; WBC, white blood cell; PD, peritoneal dialysis; CCPD, continuous cycling peritoneal dialysis; CVA, cerebrovascular accident. ^a^Not specified if antibiotics were intraperitoneal or intravenous. No duration of therapy reported.

	Reference	Age	Gender	Dialysis Modality	Culture Source	Bacteremia	Treatment	Outcome
1	Alparslan et al. [12]	12	M	CAPD	Peritoneal effluent	No	IP meropenem + TMP-SMX ×30 days	Readmitted for peritonitis; transitioned to HD
2	Quintela Obregon et al. [13]	50	F	CAPD	Peritoneal effluent; also grew Pseudomonas	No	IP imipenem + IV ciprofloxacin ×19 days	Clinical worsening and variable peritoneal WBCs; removal of PD catheter
3	Sepe et al. [14]	71	M	CCPD	Peritoneal effluent	No	IP gentamicin ×7 days	Clinical improvement with therapy; cultures to identify source of colonization were unsuccessful
4	Rihova et al. [15]	51	M	CAPD	Peritoneal effluent	No	Meropenem and amikacin^a^	Had exploratory laparotomy to confirm no bowel perforation. Clinically improved with antibiotics and retraining, with no further peritonitis episodes at follow-up
5	Esteban et al. [16]	79	F	CAPD	Peritoneal effluent	No	IP ofloxacin ×14 days	Clinical improvement with therapy; died of CVA two months after episode of peritonitis
6	Atkinson et al. [17]	55	F	CAPD	Peritoneal effluent	No	None	Peritoneal culture positive but no leukocytes; treatment deferred and symptoms improved
7	Present case	67	M	CCPD	Peritoneal effluent	No	Oral ciprofloxacin ×3 weeks	Infection cleared; retrained on proper technique but had repeat peritonitis with same organism and transitioned to HD

## Conclusions

This case underscores the importance of adequate patient education in home PD management and can inform choice of antibiotics for future occurrences. We also emphasize the significance of organism identification in PD peritonitis to most appropriately guide management in this patient population.
